# Supplementary material for: Bidirectional and context-dependent changes in theta and gamma oscillatory brain activity in noradrenergic cell-specific Hypocretin/Orexin receptor 1-KO mice
Source: Sci Rep. 2018 Oct 19;8:15474. doi: 10.1038/s41598-018-33069-8 (PMC6195537; doi:10.1038/s41598-018-33069-8)
Supplement: Supplementary file 1 — Supplementary Information [file 41598_2018_33069_MOESM1_ESM.pdf]

Supplementary Information

Title: Bidirectional and context-dependent changes in theta and gamma oscillatory brain activity in noradrenergic cell-specific *Hypocretin/Orexin receptor 1*-KO mice

Sha Li[a], Paul Franken[b], and Anne Vassalli[a*]

[a]Department of Physiology, University of Lausanne, CH-1005 Lausanne, Switzerland;
[b]Center for Integrative Genomics, University of Lausanne, CH-1015 Lausanne, Switzerland.

* To whom correspondence should be addressed. (anne.vassalli@unil.ch)

Supplementary Figures S1-S6 1
Suppl. Methods. 8
Suppl. Table 1. 9
Suppl. Table 2. 10

Supplementary Figures S1-S6

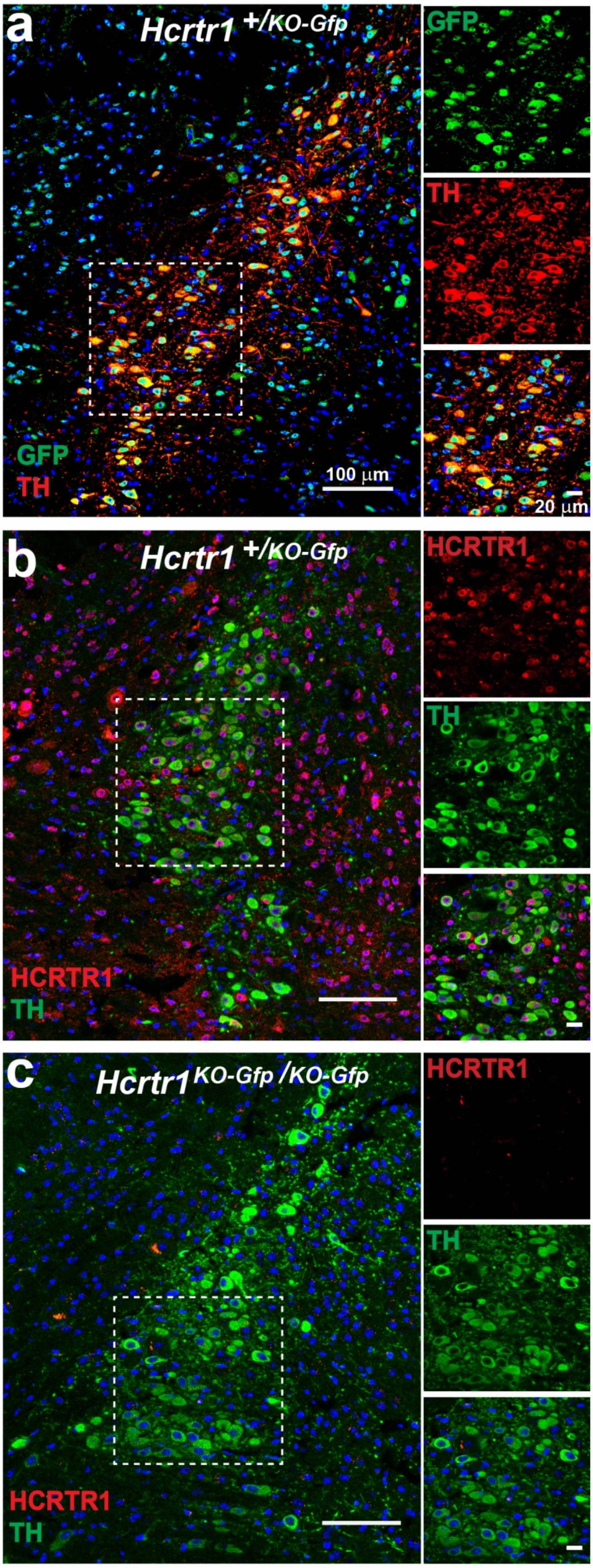

Fig. S1. CRE/loxP recombination of the floxed *Hcrtr₁* allele within the germ-line creates a GFP-reporter and constitutive KO mouse line. *Hcrtr*$_1^{flox/flox}$ mice were mated with mice harboring a Cre transgene expressed in the early embryo (*TgEIIa-Cre,* Lakso et al., 1996; see Methods) in order to transmit the CRE-recombined allele into the germ-line, and generate mice with the *KO-Gfp* allele (see Fig. 1) in all cells. (**a-c**) Representative confocal images of the locus coeruleus (LC) of mice of the indicated genotype. Noradrenergic (NA) neurons are stained with a tyrosine hydroxylase antibody (TH^+). Costaining with a GFP (a), or HCRTR1 (b,c) antibody is shown. (**a**) A heterozygous *Hcrtr*$_1^{+/KO\text{-}Gfp}$ mouse displays GFP immunoreactivity in nearly all LC TH^+ neurons (see text for cell counts). However, in contrast to CKO mice, GFP expression is not NA cell-specific, and thus not all GFP^+ cells are TH^+. CRE/loxP recombination brings a *Gfp* open reading frame in the 5' untranslated region of the *Hcrtr1* gene in lieu of the *Hcrtr1* coding sequences, thus all cells with an active *Hcrtr1* gene promoter are expected to express GFP from the *KO-Gfp* allele (Vassalli et al., 2015). (**b**) The *Hcrtr*$_1^{+/KO\text{-}Gfp}$ mouse shows HCRTR1 immunoreactivity, owing to the WT allele. In contrast, a *Hcrtr*$_1^{KO\text{-}Gfp/KO\text{-}Gfp}$ mouse lacks HCRTR1-immunoreactivity (**c**). The homozygous *Hcrtr*$_1^{KO\text{-}Gfp/KO\text{-}Gfp}$ mouse lacks HCRTR1 antibody staining within LC TH^+ cells, as well as outside the LC. The later distinguishes *Hcrtr*$_1^{KO\text{-}Gfp/KO\text{-}Gfp}$ (constitutive KO) mice from CKO mice, which lack HCRTR1 in LC TH^+ cells also, but exhibit HCRTR1-immunoreactive, TH-negative cells in areas that surround the LC nucleus (see Fig. 1e). The size indicated for scale bars in (a) applies also to (b-c).

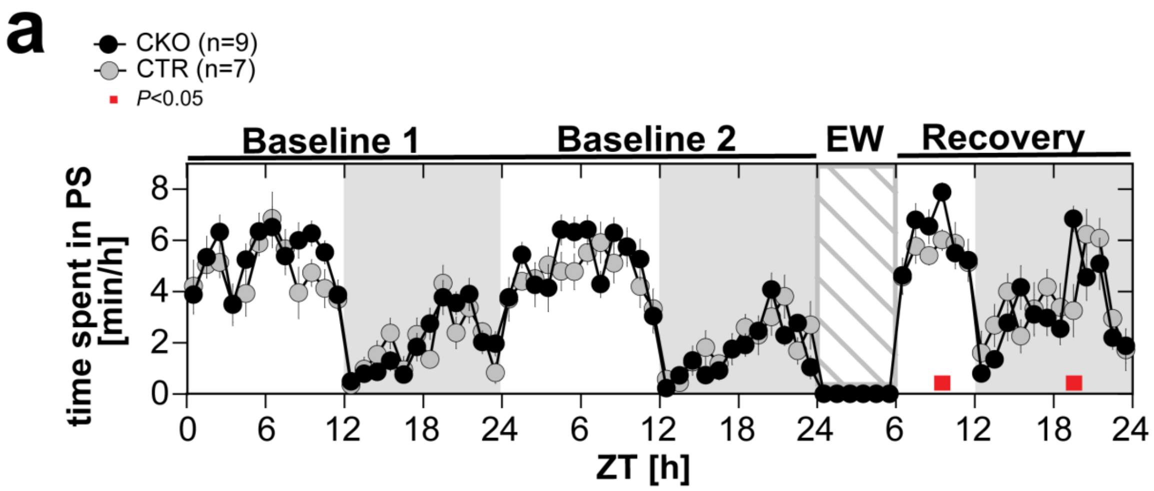

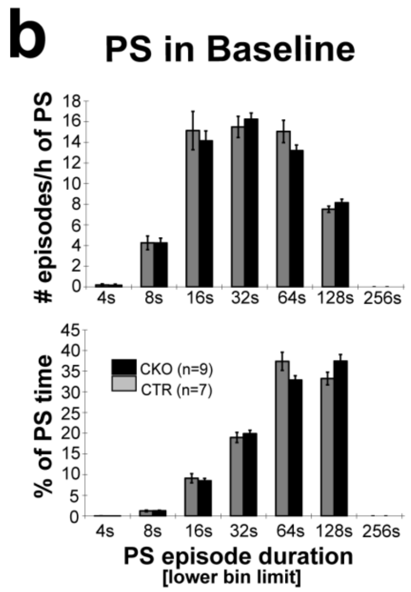

c **PS during Recovery**

		Total PS Time [min]	PS Episodes	
			Number	Mean Duration [sec]
RECOVERY ZT6-8	CKO	11.4 ± 1.2	8.6 ± 0.6	78.1 ± 7.3
	CTR	10.3 ± 0.8	8.0 ± 0.7	79.6 ± 6.8
RECOVERY ZT8-10	CKO	14.4 ± 0.5 **	10.4 ± 0.6	84.7 ± 5.6 *
	CTR	11.4 ± 0.4	10.9 ± 0.9	64.9 ± 4.1
RECOVERY ZT10-12	CKO	10.7 ± 1.1	9.0 ± 0.8	70.9 ± 3.2
	CTR	11.0 ± 1.1	10.1 ± 1.4	69.3 ± 8.7

Fig. S2. Episodes of paradoxical sleep (PS) tend to be longer in *Hcrtr1$^{Dbh\text{-}CKO}$* mice compared to *Hcrtr1$^{Dbh\text{-}CTR}$* littermates. (**a**) Hourly values (±SEM) of time spent in PS across recording days 1-3 (see Fig. 2) for CKO (●, n=9) and CTR mice (◯, n=7). This timecourse differs significantly between the two genotypes (two-way ANOVA, genotype X time interval interaction, P=0.01). ■ indicate hours of significant genotype differences; t-test, P< 0.05). Recovery in light phase ZT6-12 is further analyzed in (c). Grey areas indicate the dark phase. The striped area indicates the 6h enforced waking (EW) period. (**b**) Distribution of PS in episodes of various duration categories in baseline conditions (days 1-2). (***Top***) Average number of PS episodes of each duration per hour of PS. (***Bottom***) Percentage of PS spent in episodes of each duration. CKO mice display a right shift in the distribution of percent time spent in PS across episode duration categories (two-way ANOVA, genotype X episode duration interaction, P<0.002). (**c**) Analysis of PS episodes' number and duration in the 6h of light phase recovery (ZT6-12) following EW. CKO expressed a 26% increase in PS time over CTR mice in recovery ZT8-10, the period of peak PS rebound (CKO: 14.4±0.5 min vs CTR: 11.4±0.4 min; t-test P=0.0005, **). This is due to a 20-s lengthening of mean PS episode duration in CKOs (+31%) (CKO: 84.7±5.6 s vs CTR: 64.9±4.1 s; t-test, P=0.018, *), while episode number is unaffected (-5%; t-test, P=0.691).

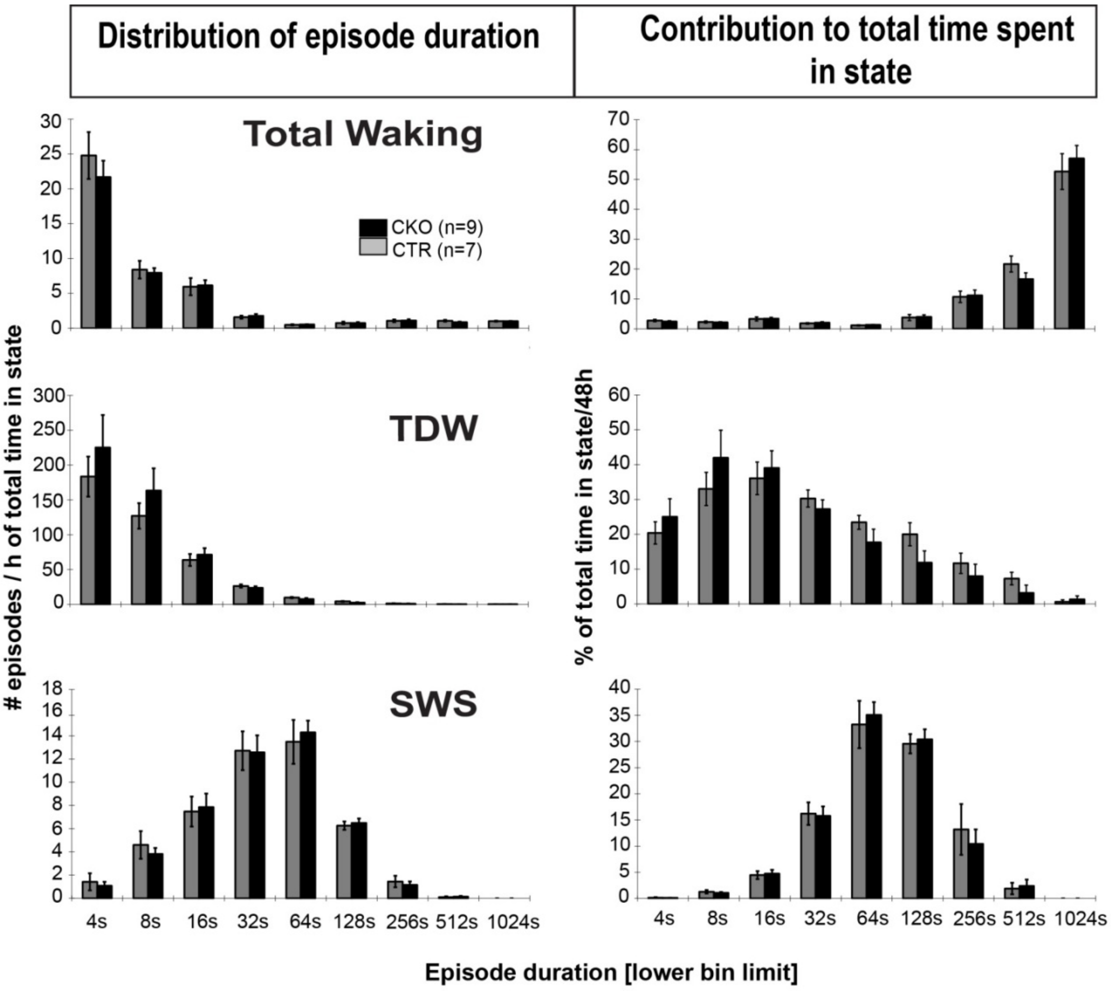

Fig. S3. Distribution of all-waking (W), theta-dominated waking (TDW), or SWS episodes in undisturbed conditions (baseline days 1-2) in *Hcrtr1*$^{Dbh\text{-}CKO}$ (n=9) and *Hcrtr1*$^{Dbh\text{-}CTR}$ mice (n=7). (***Left***) The average number of episodes per hour spent in each respective state is shown for each of nine categories of episode duration. (***Right***) The percentage of total time in each state that is spent in bouts of each duration category is depicted.

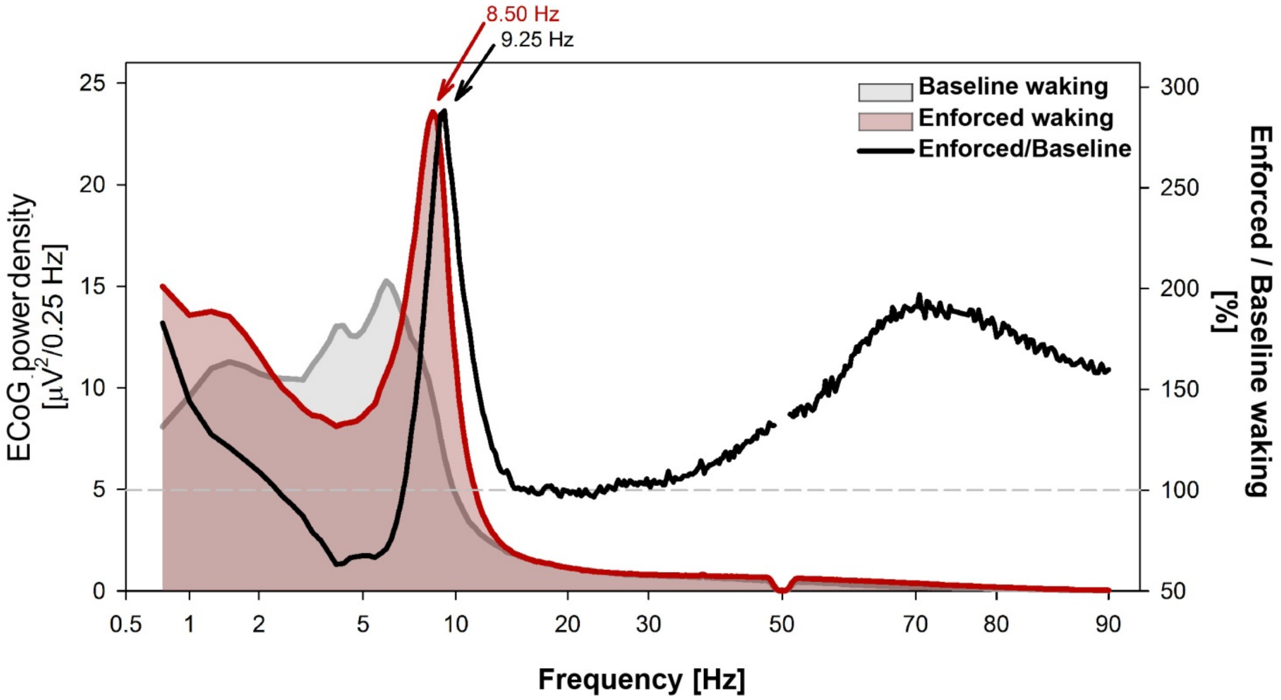

Fig. S4. Interpreting ECoG spectral ratios: maxima in power spectral density ratios do not represent peaks of oscillatory activity. To contrast two power spectra, or to express a spectral profile relative to another spectral profile (e.g. EW spectra vs. baseline waking spectra), the ratio of the two spectra should be interpreted with caution: a peak in this spectral ratio represents a frequency at which the two spectra differ maximally, and should not be confused with the peak of the oscillations on which the two spectra being compared are based. To illustrate this, the mean EW' oscillatory activity spectrum of *Hcrtr1*$^{Dbh\text{-}CTR}$ mice (n=7; red), and the baseline spectrum to which it is contrasted, i.e. baseline waking in light phase last 4 h (ZT8-12) (grey), are shown. EW exhibits a sharp θ rhythm, of maximal power at 8.5 Hz. The ratio between these two spectra (black) features a peak at 9.25 Hz. This is the frequency at which the two spectra differ the most. It does not evidence the occurrence of 9.25 Hz brain oscillations.

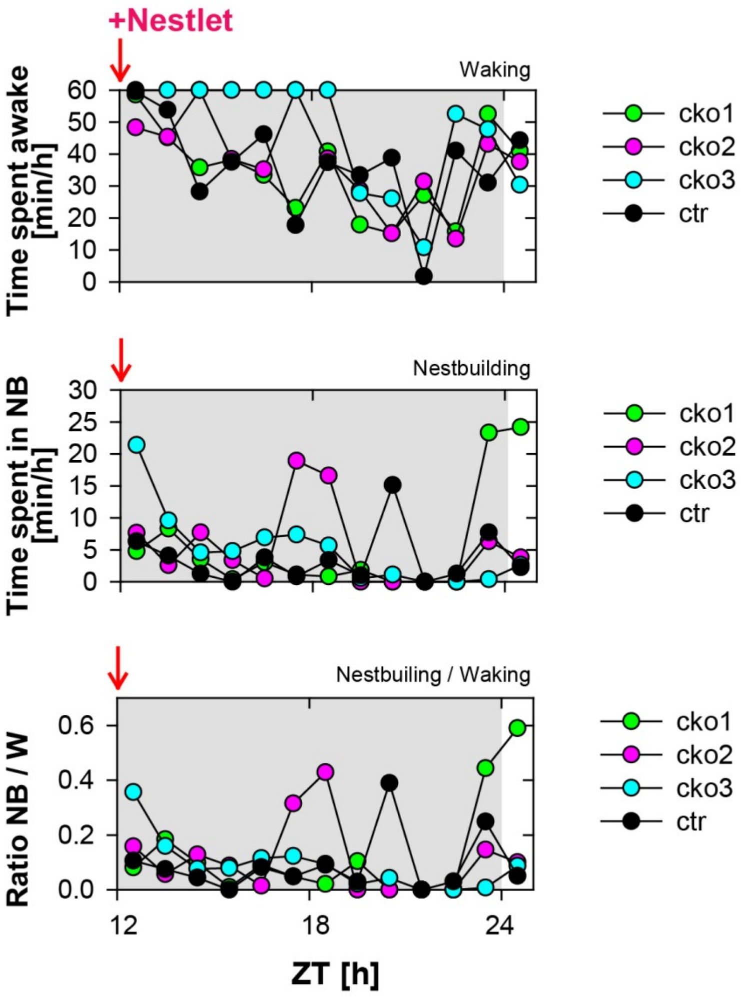

Fig. S5. Consolidated nestbuilding activity tends to mostly occur in the 2nd half of the night, in particular in the last 2 hours (ZT22-24), preceding the major sleeping period. Nestbuilding activity (NB) was analyzed by IR video imaging across the night following addition of a Nestlet at dark onset (red arrow) in four mice (3:1, CKO:CTR). Episodes in which the mouse manipulated or moved around nest material (using paw or mouth) lasting a minimal of 3 epochs (≥12 s) were scored as 'NB'. Total time [min/h] spent awake (Top), in NB behavior (Middle), or the time in NB behavior per waking h (Bottom), are plotted across time.

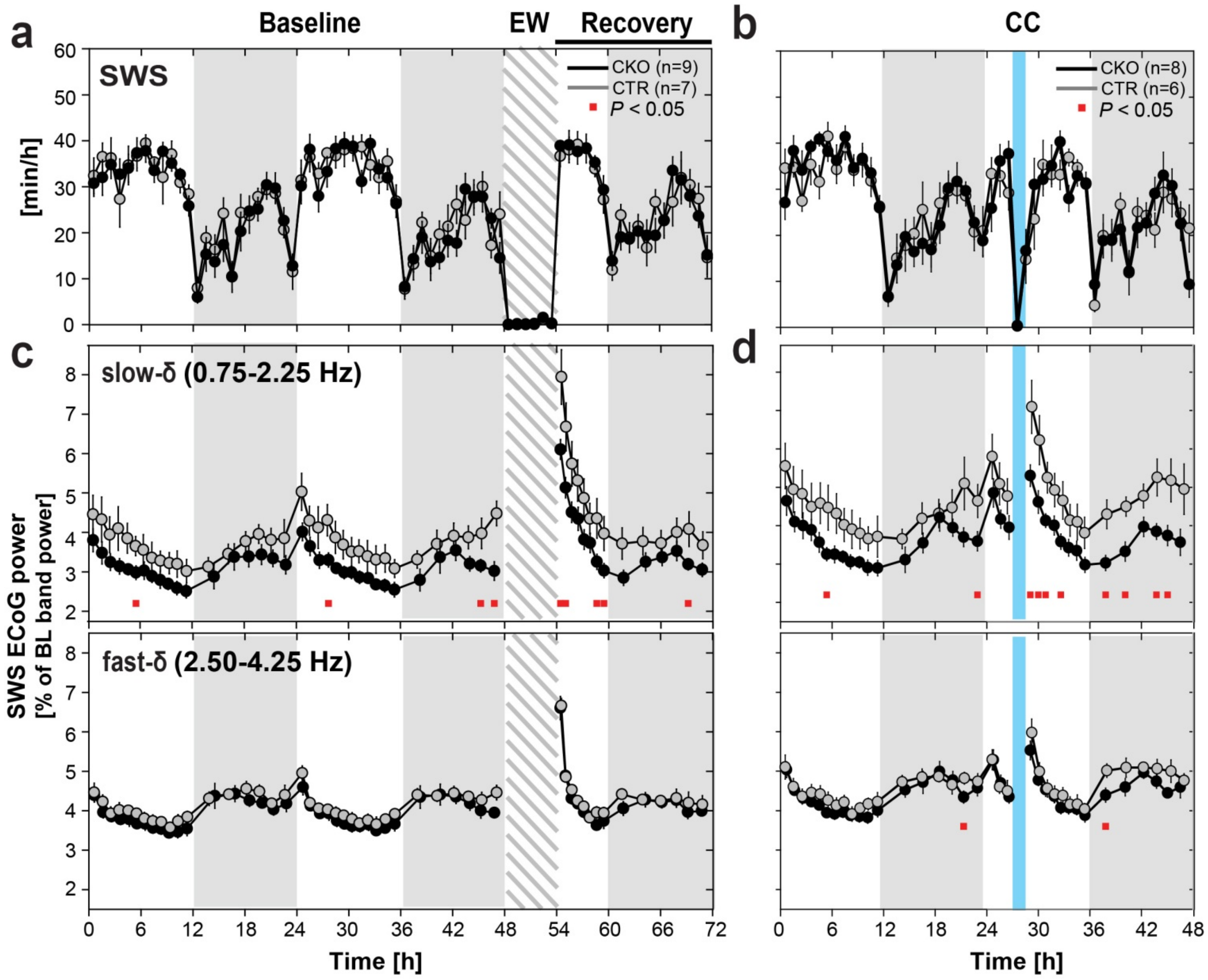

Fig. S6. Dynamics of slow-δ (δ1: 0.75-2.25 Hz) and fast-δ (δ2: 2.5-4.25 Hz) power in SWS of *Hcrtr1*$^{Dbh\text{-}CKO}$ and *Hcrtr1*$^{Dbh\text{-}CTR}$ mice after different waking conditions. Time spent in SWS [min/h] across days 1-3 (comprising enforced waking, EW) (**a**), or days 8-9 (comprising cage change, CC) (**b**). Timecourses of ECoG δ1 (**c** *Top* and **d** *Top*), and δ2 (*Bottom*) power are expressed as % of baseline total power calculated for each mouse across all frequencies and all states (see Methods). δ1 CKO values are inferior to CTR values at all times, and most significantly so in SWS following major active waking periods, such as in baseline dark phase, and after EW (**c** *Top*), or after CC-induced waking (**d** *Top*). Red bars indicate significant genotype differences (post-hoc two-sided t-tests, $P<0.05$).

Suppl. Methods.

Mice were in a mixed *C57BL/6NTac* X *C57BL/6J* background. We analyzed mice from both these inbred strains. It is noteworthy that we found the two strains to differ in sleep/wake-associated variables. Notably, *C57BL/6NTa*c mice spent significantly less time awake, and more time in SWS, and in PS, than *C57BL/6J* mice (unpaired two-tailed Student's *t* test; wake: *P*= 0.02; SWS: *P*= 0.048; PS: *P*=0.03). The distribution of behavioral states was (wake; SWS; PS): 50.57±1.24 %; 43.74±1.14 %; 5.69± 0.27 % for *C57BL/6NTac* mice (n=8), and 54.86±1.14 %; 40.25±1.12 %; 4.89± 0.21 % for *C57BL/6J* mice (n=11).

Suppl. Table 1.

Expression of behavioral states across recording days1-3 (24 h baseline, and 18-h 'recovery' following 6-h enforced wakefulness, EW) in *Hcrtr1*$^{Dbh\text{-}CKO}$ (*CKO*, n=9) and *Hcrtr1*$^{Dbh\text{-}CTR}$ (*CTR*, n=7) littermate mice.

Time spent in waking (W), SWS, and PS [hours] (mean ± SEM).

Baseline (days1+ 2)				Recovery after 6-h EW (day 3)			
	W	SWS	PS		W	SWS	PS
**24-h**				**18-h Recovery**			
CTR	11.70 ± 0.42	10.97 ± 0.39	1.33 ± 0.08	*CTR*	14.63 ± 0.28	8.13 ± 0.24	1.24 ± 0.06
CKO	12.01 ± 0.41	10.58 ± 0.38	1.41 ± 0.05	*CKO*	14.65 ± 0.40	8.10 ± 0.36	1.25 ± 0.06
**12-h Light (ZT0-12)**				**6-h Light (ZT6-12)**			
CTR	4.17 ± 0.18	6.88 ± 0.17	0.95 ± 0.05	*CTR*	1.90 ± 0.11	3.55 ± 0.10	0.40 ± 0.03
CKO	4.18 ± 0.17	6.77 ± 0.15	1.05 ± 0.04	*CKO*	1.75 ± 0.12	3.64 ± 0.10	0.61 ± 0.03
**12-h Dark (ZT12-24)**				**12-h Dark (ZT12-24)**			
CTR	7.53 ± 0.30	4.09 ± 0.27	0.38 ± 0.04	*CTR*	6.76 ± 0.27	4.55 ± 0.23	0.69 ± 0.05
CKO	7.83 ± 0.28	3.81 ± 0.26	0.37 ± 0.03	*CKO*	6.95 ± 0.08	4.42 ± 0.26	0.64 ± 0.04

Suppl. Table 2.

Peak frequency of the θ oscillatory rhythm expressed by *Hcrtr1*$^{Dbh\text{-}CKO}$ (*CKO*, n=9) and *Hcrtr1*$^{Dbh\text{-}CTR}$ (*CTR*, n=7) mice while awake in different behavioral contexts.

TDW, theta-dominated-waking. *, two-tailed t-test, *P*<0.05.

	Theta Peak Frequency [Hz] (mean±SEM)		
	CKO	***CTR***	**Genotype difference *P*-value**
Baseline dark phase Active Wake (TDW) (ZT12-18)	8.28±0.12	8.57±0.15	*0.27*
Enforced wakefulness (ZT0-6)	8.14±0.09	8.64±0.09	*0.002* *
Cage change-induced wakefulness (ZT3-4)	8.06±0.19	8.50±0.16 *	*0.059*
